# Designed Natural Spaces: Informal Gardens Are Perceived to Be More Restorative than Formal Gardens

**DOI:** 10.3389/fpsyg.2016.00088

**Published:** 2016-02-11

**Authors:** Elyssa Twedt, Reuben M. Rainey, Dennis R. Proffitt

**Affiliations:** ^1^Department of Psychology, St. Lawrence UniversityCanton, NY, USA; ^2^Department of Landscape Architecture, University of VirginiaCharlottesville, VA, USA; ^3^Department of Psychology, University of VirginiaCharlottesville, VA, USA

**Keywords:** perceived restoration, restorative environments, garden design, natural spaces, built spaces

## Abstract

Experimental research shows that there are perceived and actual benefits to spending time in natural spaces compared to urban spaces, such as reduced cognitive fatigue, improved mood, and reduced stress. Whereas past research has focused primarily on distinguishing between distinct categories of spaces (i.e., nature vs. urban), less is known about variability in perceived restorative potential of environments *within* a particular category of outdoor spaces, such as gardens. Conceptually, gardens are often considered to be restorative spaces and to contain an abundance of natural elements, though there is great variability in how gardens are designed that might impact their restorative potential. One common practice for classifying gardens is along a spectrum ranging from “formal or geometric” to “informal or naturalistic,” which often corresponds to the degree to which built or natural elements are present, respectively. In the current study, we tested whether participants use design informality as a cue to predict perceived restorative potential of different gardens. Participants viewed a set of gardens and rated each on design informality, perceived restorative potential, naturalness, and visual appeal. Participants perceived informal gardens to have greater restorative potential than formal gardens. In addition, gardens that were more visually appealing and more natural-looking were perceived to have greater restorative potential than less visually appealing and less natural gardens. These perceptions and precedents are highly relevant for the design of gardens and other similar green spaces intended to provide relief from stress and to foster cognitive restoration.

## Introduction

Over 2.75 million people visit the United States National Parks annually (nps.gov); conservation groups such as the Sierra Club and the National Audubon Society fight to protect wildlife and their natural habitats; and people pay premium prices for waterfront or garden view properties. These behaviors suggest an inherent attraction to “nature” and an intuition that spending time in “natural” spaces is beneficial to well-being. Natural spaces, as we employ the term for this study, are outdoor spaces characterized by an abundance of plants and other elements of natural ecosystems (Kaplan et al., 1998). Natural spaces include wilderness areas and designed spaces such as parks and gardens.

It has been suggested that humans have an automatic positive emotional response and psychological preference for natural environments over urban environments, due to the fact that humans and pre-*Homo sapiens* evolved in and adapted to natural environments for millions of years (Kaplan and Kaplan, 1989; Ulrich, 1995; Grinde and Patil, 2009). Biologist E. O. Wilson named this the *biophilia hypothesis* to describe human's predilection for all living things. According to the *biophilia hypothesis*, humans depend on nature for “aesthetic, intellectual, cognitive and even spiritual meaning and satisfaction” (Kellert and Wilson, 1995, p. 20). While built environments—such as private homes, public buildings, and well-designed contemporary urban landscapes—also have the potential to enhance well-being (Kaplan et al., 1993; Scopelliti and Giuliani, 2004; Karmanov and Hamel, 2008), natural spaces might be particularly conducive to restorative experiences.

For many people, taking a trip to visit a large natural wildlife area such as a national park provides an escape from the fatigue of everyday routine. These prolonged interactions with nature have been linked to increased creativity and cognitive recovery (Hartig et al., 1991; Atchley et al., 2012). However, more often, our contact with nature comes in smaller doses from nearby spaces such as city parks or gardens. The importance of having easy access to nature, even in the midst of a city setting, was emphasized by Olmsted (1865), one of the designers of Central Park in New York City. Olmsted stated, “It is a scientific fact, that the occasional contemplation of natural scenes of an impressive character…is favorable to the health and vigor of men.” Areas of Central Park look very natural, yet are as built as anything else in New York. Parks and gardens make for unique test cases when studying restorative environments because, despite being categorized as natural spaces, they are often designed and built by landscape architects, architects, and other design professionals, as opposed to being the natural wilderness areas found in protected national parks.

Focusing on these unique test cases, the question addressed in the current study is “What specific characteristics of designed natural spaces, particularly gardens, relate to perceived restorative potential?” Perceived restorative potential is defined as individuals' judgments of the degree to which an environment might afford recovery of resources. For example, if a person perceives a space to have high restorative potential, then the person expects that spending time in that space will result in psychological, emotional, or physiological recovery. Perceived restorative potential differs from psychological restoration, which is an actual improvement to individuals' well-being (e.g., recovery of cognitive resources, increased positive mood, reduced stress).

Gardens manifest a wide variety of design expressions determined by the cultural values of their creators and their response to the particular landforms, geology, climate, and ecosystems of their locations. While opinions vary among scholars as how best to characterize these variations, one prevalent system of classification organizes gardens on a typological spectrum ranging from “formal” or “geometric” to “informal” or “naturalistic.” We have used this system in our study because it is useful for describing the distinguishing visual properties of gardens and is a familiar one to lay persons. “Formal” gardens tend to be organized with bilateral or radial symmetry, characterized by the use of elements exhibiting clear Euclidian geometry, such as straight lines, circles, arches, sharp angles, and axes. Such gardens often, but not always, contain numerous architectural elements, including pavilions, fountains, walls, and sculptures. They typically transform the natural topography of their sites into flat or inclined planes. Their plant palette tends to use more exotic plant species rather than ones native to their region, and they contain plants pruned into geometric or animal shapes rather than exhibiting their natural forms. Seventeenth-century French and Italian Renaissance gardens and their similar expressions in residential gardens today are typical examples of “formal” garden design. In contrast, “informal” or “naturalistic” gardens tend to leave the terrain of their sites unaltered, or sculpt it in graceful curvilinear shapes. They generally contain fewer architectural elements, use more native plant species, and express plants in their more natural habits of growth. Their water features resemble more natural forms, such as brooks and ponds. Sculpture is downplayed as are axes, symmetries, and other forms of strong Euclidian geometry. In their most pronounced form “informal” gardens are hardly distinguishable from natural ecosystems and show little apparent sign of human intervention although they are carefully wrought works of art. Typical examples are nineteenth-century English and American so-called “wild gardens,” twelfth-century Japanese Stroll Gardens, and late-eighteenth-century English Landscape Gardens. However, “formal” and “informal” gardens are seldom expressed as pure types, but appear on a spectrum tending toward one or the other. The graphic examples employed in this study contain both historical and contemporary examples.

The predominant account for explaining restorative experiences is the Attention Restoration Theory (ART; Kaplan, 1995). According to this approach, restorative spaces are particularly well-suited for engaging attention effortlessly while simultaneously allowing for depleted attentional resources to replenish. A robust body of research shows that natural spaces may improve cognitive performance, enhance positive mood, reduce rumination, and reduce stress (e.g., Ulrich, 1984; Taylor et al., 2002; Berman et al., 2008; Bratman et al., 2015a,b; Pilotti et al., 2015). These conclusions are largely based on studies employing dichotomous comparisons between natural and built spaces.

Rather than focusing on this simplistic dichotomy, there are benefits to measuring variability both between and within setting types. For example, Twedt et al. (under review) asked participants to judge the perceived restorative potential of environments that ranged from completely natural to completely built, including a sample of spaces that included a mixture of natural and built elements. Participants provided estimates of visual appeal, naturalness, and the presence of people for each environment, and reported their energy levels and mood, which served to indicate participants' current need-for-restoration. Need-for-restoration is an assessment of the degree to which an individuals' resources are depleted and therefore would most benefit from spending time in a restorative environment. Greater visual appeal, more natural elements as opposed to man-made elements, and fewer people present related to higher perceived restorative potential. Furthermore, people who reported feeling fatigued showed a lower preference to spend time in built and mixed spaces than people who reported feeling energized. Interestingly, participants with high arousal levels reported higher preferences for spending time in built spaces than participants with low arousal levels. This study highlighted the dynamic interaction between person and setting. People are sensitive to the variability of perceived restorative potential within setting type. Not all natural scenes are perceived to be restorative and not all built environments are perceived to be depleting (see also Kaplan et al., 1993; Scopelliti and Giuliani, 2004; Karmanov and Hamel, 2008; Martínez-Soto et al., 2014 for examples of how built environments vary on restorative potential).

Most studies of designed natural spaces have sought to identify the features of small urban parks that relate to perceived restorative potential. People perceive that urban parks have a higher likelihood of restoration when features such as large spaces, water, plants, and grass are present, whereas traffic, city buildings, and built surfaces are hidden or minimized (Nordh et al., 2009, 2011; Nordh and Østby, 2013). People also judge parks with greater biodiversity—more variation in plants and flowers—to have greater restorative potential (Carrus et al., 2015). The amount of people present in a park also impacts restorative potential; too few people in a park can signal an unsafe environment, whereas too many people in a park can indicate crowding (Nordh et al., 2011). Van den Berg et al. (2014) found that self-reported restoration improved more for park settings relative to urban settings and that recovery depended on perceived naturalness of the park settings.

Less work has focused specifically on gardens. Ivarsson and Hagerhall (2008) showed that the Perceived Restorativeness Scale—a validated measure of perceived restorative potential—can be used to distinguish between gardens that vary in the amount of natural and built elements present. Participants perceived a larger, more natural garden to be more restorative than a more built, smaller garden. Their study showed that restorative potential can vary within scene category. However, only two garden exemplars were used in their study, limiting its generalizability, and highlighting the need to sample a larger and more diverse set of gardens.

Hadavi et al. (2015) asked participants to categorize ninety-three different garden and park scenes according to preference, type (e.g., natural, man-made), and what activities the spaces afford. Participants categorized gardens that were geometric and included built structures for seating to be “man-made but not natural.” Conversely, gardens with more organic designs were labeled as “natural and man-made.” These descriptions are somewhat consistent with our formal and informal garden exemplars, respectively. Interestingly, favorite spaces among their participants included both gardens that appeared man-made and gardens that appeared more natural, rather than showing a pure bias for natural-looking gardens.

We conducted a study to assess what qualities make a garden appear to be restorative, using a sample of 40 gardens varying with regard to design informality. We focused on broader experiential aspects of gardens, including their visual appeal, perceived naturalness, and apparent informality of design rather than individual physical features such as water or flowers. These are qualities that will be perceived more as a comprehensive whole rather than as isolated specific elements.

Participants viewed images of designed gardens. Gardens varied in the degree to which each was designed to appear more formal or more informal. For each image, participants judged the perceived restorative potential, visual appeal, degree of naturalness and degree of design informality. We predicted that participants would perceive more informal gardens to have higher restorative potential than more formal gardens, given that informal gardens tend to be more natural and devoid of built elements than formal gardens.

Second, we predicted that perceived restorative potential would be higher for more visually appealing and naturalistic gardens. If this hypothesis was supported, we aimed to explore whether visual appeal mediated the relationship between design informality and perceived restorative potential. That is, are informal gardens perceived to be more restorative because they are more visually appealing? This prediction is partially motivated by a previous study that found participants' perceived stress reduction was mediated by perceived attractiveness of a hospital room that did or did not contain plants (Dijkstra et al., 2008). Furthermore, past research often compares the perceived restorative potential of visually appealing natural spaces to visually unappealing urban spaces introducing a potential confound for which future studies could control. Because informal gardens tend to be more naturalistic by design, we inferred that informal gardens might inherently be more visually appealing than formal gardens which in turn could account for differences in perceived restorative potential.

In contrast to prior research, this study sampled a much larger and more demographically diverse population. Thus, we were able to conduct exploratory analyses that addressed whether variability in perceived restorative potential of gardens is moderated by demographic characteristics such as participants' self-reported age, sex, education, income, and residential experience.

These anticipated findings would be consistent with prior work investigating the positive relationship between naturalness and perceived restorative potential of spaces consisting of primarily natural ecosystems with little human intervention, while extending this account to designed natural spaces such as gardens.

## Methods

### Participants

We aimed to recruit 350 participants from Mechanical Turk—an online marketplace supported by Amazon.com to conduct online research. Three hundred and fifty-three people completed the study for $1.00 payment (participants could have completed the study but never finalized their submission through Mechanical Turk, resulting in a larger sample than originally intended). Fifty-eight participants were excluded from the data analysis because they either tried to complete the study twice or had previously participated in a similar study. Therefore, 295 participants were retained for data analysis (129 men, *M*_age_ = 33.67; *SD*_age_ = 12.15; range: 18–82). The ethnic diversity of the sample included 82.7% White, 6.4% Black, 4.7% Hispanic or Latino, 4.1% Asian, 1% Middle Eastern, and 1% Other. The majority (54.9%) of participants had earned a 2-year college degree or higher and 35.9% had completed at least some college. Thirty-six percent of participants earned under $50,000 annual household income, with income ranging from less than $35,000 to more than $250,000. This study was carried out in accordance with the recommendations of the Institutional Review Board at the University of Virginia and all participants gave informed consent.

### Materials

**Figure 1:**
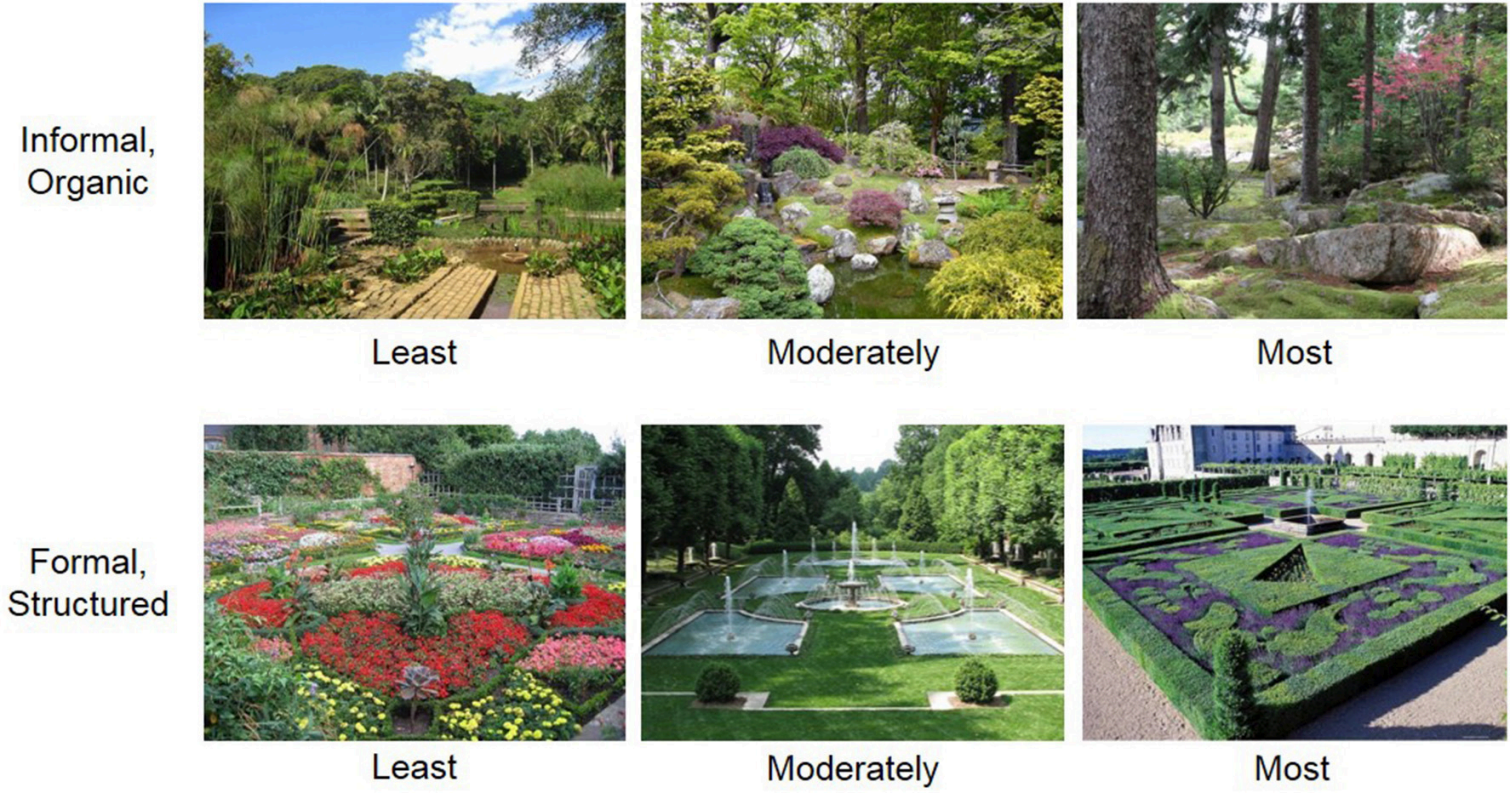
**Example stimuli**. Gardens ranged from informal/organic (top) to formal/structured (bottom), and from least informal/formal (left) to most informal/formal (right).

Participants viewed 40 images of different gardens (see Figure 1 for examples). Images were collected from a local professor of landscape architecture (i.e., the second author of this paper, RR) with over 30 years of experience in teaching the history of garden design. The images were captured with a wide-angle lens to include as comprehensive a view as possible of each garden example. Images were scaled to 400 × 600 pixels and were presented horizontally in color. The primary goal in curating an image set was to include gardens that ranged from very informal to very formal on a continuous dimension.

To select the final image set, RR rated a larger database of gardens using the following criteria. First, RR confirmed that each garden could be classified as either formal or informal. Second, he ranked each image on the degree to which that image exemplified a description of formal or informal design. That is, each image classified as more formal received a ranking between 1 (least formal) and 5 (most formal), in half-point increments. Similarly, each image classified as more informal received a ranking between 1 (least informal) and 5 (most informal), in half-point increments. Two to three images were selected from each rank to ensure that the gardens chosen represented a range of informality. In the final image set, half of the gardens could be broadly classified as ranging from very informal to a little informal and half of the gardens could be broadly classified as ranging from very formal to a little formal. The stimulus set can be viewed at osf.io/xi42e.

### Design

This study used a within-participants design. Each participant viewed all 40 images (20 more formal/structured gardens and 20 more informal/organic gardens). Participants rated each environment on four dimensions: perceived restorative potential (PRP), visual appeal, naturalness, and the degree to which the environment is formal/structured vs. informal/organic, for a total of 160 trials. Ratings for each dimension were blocked such that participants rated all images on one dimension (e.g., visual appeal), then rated all images on another dimension (e.g., naturalness), until all four dimensions had been rated. Block order and image order within block were randomly presented.

### Procedure

Participants completed the study using Qualtrics, an online survey design program. Participants first provided demographic information including their age, sex, race, zip code for current residence, highest level of completed education, and household income.

Next, participants viewed a preview of all 40 images that they would later be asked to rate. Each image appeared for 2 s in a randomized order. The image preview ensured that participants had a sense of the entire range of images that they would rate, which would presumably help participants calibrate their ratings across the image set.

For the image rating task, participants completed four blocks of trials in which they rated each image on perceived restorative potential, informality, visual appeal, and naturalness. The instructions and rating scale for each block of trials were as follows:

1. Perceived Restorative Potential: Recall a time when you felt overwhelmed, stressed, and anxious. Reflect on how you felt in that moment and put yourself in that mindset. Continue to reflect on this mindset as you view the pictures and imagine yourself in each environment. For each of the images presented, rate the degree to which you think being in that environment would be a good place for you to take a break and make you feel less stressed and anxious, using a scale from 0 (Not at all a good place for a break) to 100 (Very much a good place for a break).
2. Informality: For each of the images presented, rate the degree to which that environment is formal (i.e., structured, symmetrical, geometric) as opposed to informal (i.e., organic, naturalistic, asymmetrical), using a scale from 0 (Completely Formal/Structured) to 100 (Completely Informal/Organic).
3. Visual Appeal: For each of the images presented, rate how visually appealing that environment is to you, on a scale from 0 (Not at all Visually Appealing) to 100 (Very Visually Appealing).
4. Naturalness: For each of the images presented, rate the degree to which that environment contains nature, as opposed to man-made/built elements, using a scale from 0 (Completely Man-Made/Built) to 100 (Completely Natural).

**Figure 2:**
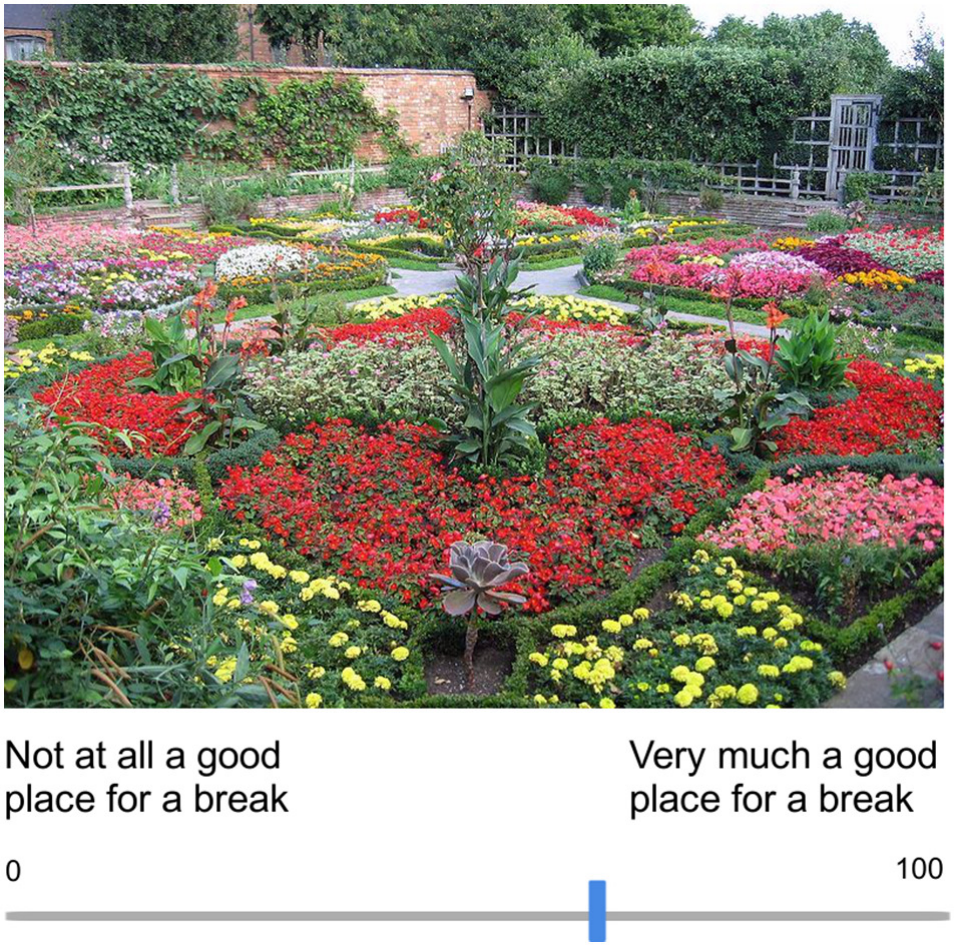
**Example perceived restorative potential trial**.

Participants made their responses by moving a slider along a continuous scale from 0 to 100 (see Figure 2). Before beginning the rating task, participants completed one practice trial to familiarize themselves with the task. Participants had to move the slider along the sliding scale before advancing to the image rating trials.

## Results

Data and analyses are available at https://osf.io/ucn86/ and https://osf.io/za8q7/, respectively. Descriptive statistics for mean image ratings are provided in Table 1. Average ratings for mean informality, as provided by participants, closely matched our *a priori* rankings of image informality suggesting that we successfully sampled gardens ranging from very formal to very informal.

**Table 1:** **Descriptive statistics for image ratings averaged across participants**.

***a priori ranking***	**Image rank**	**Informality**	**Naturalness**	**PRP**	**Visual appeal**
Most formal	1	7.48	11.88	34.48	50.35
	2	10.24	14.56	35.70	46.78
	3	11.40	14.63	40.04	48.05
	4	6.99	11.73	39.75	54.65
	5	11.59	11.36	40.23	46.89
	6	10.31	17.59	35.58	46.30
	7	10.53	14.97	39.78	50.49
	8	15.73	21.37	50.11	51.10
	9	12.01	19.31	38.86	45.37
	10	8.19	13.28	48.07	58.91
	11	11.38	16.81	56.65	61.31
	12	11.36	14.11	47.77	48.71
	13	33.38	34.56	27.89	27.57
	14	32.66	33.98	49.27	50.17
	15	34.39	34.24	61.51	60.48
	16	31.22	34.57	48.86	51.54
	17	26.07	23.01	55.98	59.61
	18	31.12	32.49	47.62	52.88
	19	35.80	41.95	63.34	70.63
	20	48.54	48.19	48.45	45.86
	21	23.63	26.37	56.78	54.10
	22	53.01	47.32	61.98	54.18
	23	33.85	32.89	65.68	67.69
	24	64.11	58.12	75.90	74.76
	25	67.77	59.63	63.84	59.97
	26	50.62	46.99	51.64	51.37
	27	63.70	55.69	76.96	74.21
	28	74.33	73.16	79.70	72.11
	29	72.62	67.12	57.58	60.53
	30	63.99	57.73	63.76	53.77
	31	62.73	59.68	71.18	65.67
	32	72.12	61.00	73.13	70.25
	33	63.24	58.46	82.16	79.17
	34	60.36	55.99	67.56	64.35
	35	81.11	78.34	83.17	80.42
	36	66.13	59.14	82.13	80.76
	37	76.62	73.43	71.96	66.85
	38	74.84	72.13	66.55	60.49
	39	86.64	87.62	80.76	75.41
Most informal	40	90.11	91.88	82.34	72.90

*Image number corresponds to a priori ranking of garden design informality*.

*Informality: low values = more formal; high values = more informal; Naturalness: low values = more built; high values = more natural*.

### Correlations between feature ratings

We tested correlations for all pairings of the four rating dimensions using repeated-measures correlations (Bland and Altman, 1995). This method takes into account that each participant made 40 rating estimates per dimension. It provides a means to test the variation within participants rather than aggregating across images; that is, if there is an increase in one variable within a participant for a given image, is there a subsequent increase in the second variable?

Within-participants, perceived restorative potential significantly correlated with visual appeal (*r* = 0.60, *p* < 0.001), naturalness (*r* = 0.43, *p* < 0.001), and informality (*r* = 0.44, *p* < 0.001). Visual appeal significantly correlated with naturalness (*r* = 0.29, *p* < 0.001) and informality (*r* = 0.30, *p* < 0.001). Naturalness significantly correlated with informality (*r* = 0.72, *p* < 0.001); this high correlation is not surprising given that informal gardens are partially defined by naturalistic design features. These results suggest that when participants perceived a garden to be visually appealing, natural, or informal, they also perceived that garden to be more potentially restorative compared to gardens that were visually unappealing, built, or formal.

### Evaluations of gardens ranging in design informality

Because each participant rated 40 images, a multi-level model is most appropriate for all following analyses. Three separate linear mixed effects models fit by maximum likelihood estimation were used to test the relationship between informality and the outcome variables of perceived restorative potential, visual appeal, and naturalness. For all three models, participants and images were included as random factors and an unstructured covariance structure was assumed.

Perceived restorative potential, naturalness, visual appeal, and design informality were first logit-transformed. We chose a logit-transformation to correct for potential bias that is introduced into a model when variables have a constrained, u-shaped distribution (e.g., more values on the low and high ends of a distribution). For example, there were a disproportionate number of images rated as 0 and 100 on the perceived restorative potential scale relative to ratings on the middle of the scale. To logit transform, variables were first rescaled into a 0 to 1 scale. Next, a small amount of random noise was added to 0 and 1 values, because logit cannot be applied to those values. These rescaled values were then logit transformed (logit = log(x/1-x)) to create an unbounded variable.

As predicted, more informal gardens predicted higher perceived restorative potential, greater visual appeal, and more naturalness (see Table 2 for parameter estimates).

**Table 2:** **Parameter estimates predicting image evaluations from perceived design informality**.

**Rating**	**Intercept (*SE*)**	**Estimate (*SE*)**	***t*-value**	**95% CI around estimate**	**σ^2^ participant (*SD*)**	**σ^2^ image (*SD*)**
PRP	0.76 (0.17)	0.15 (0.01)	15.20	0.13, 0.17	1.89 (1.37)	0.92 (0.96)
Visual appeal	0.81 (0.14)	0.13 (0.01)	13.76	0.11, 0.15	1.56 (1.25)	0.57 (0.75)
Naturalness	−0.44 (0.21)	0.27 (0.01)	35.46	0.26, 0.29	0.89 (0.94)	1.58 (1.26)

*p < 0.001 for all tests; standard errors in parentheses*.

### Predicting perceived restorative potential

What dimensions predict the perceived restorative potential of gardens? We fit a linear mixed effects model using maximum-likelihood estimation to predict perceived restorative potential from the continuous variables of visual appeal, informality, and naturalness. All variables were first logit-transformed. We did not predict any interactions *a priori* so only main effect terms were included in the model. This model was compared to a model in which perceived restorative potential was only predicted from the intercept. In all models, participants and image stimuli were treated as random variables and we assumed an unstructured covariance structure.

Gardens that were more visually appealing, more informal, and contained more “nature” (plants and other elements of natural ecosystems) were perceived to be more restorative than less visually appealing, more formal, and more built gardens (Table 3). Visual appeal accounted for the largest amount of variance in the model. There was significant between-participant variation and between-image variation. Including visual appeal, informality, and naturalness as predictor variables improved model fit compared to the no predictor model.

**Table 3:** **Mixed effect model parameter estimates predicting perceived restorative potential**.

**INTERCEPT ONLY MODEL**				
**Fixed effects**				
Name	Estimate	*SE*	*t*-value	95% CI around estimate
(Intercept)	0.66	0.21	3.14	0.24, 1.08
**Random effects**				
Group	Name	Variance	*SD*	95% CI around SD
Participants (*N* = 295)	(Intercept)	1.90	1.38	1.27, 1.50
Image (*N* = 40)	(Intercept)	1.50	1.23	1.00, 1.56
Residual		4.27	2.07	2.04, 2.09
BIC = 51710.2				
**MAIN EFFECTS MODEL**				
**Fixed effects**				
Name	Estimate	*SE*	*t*-value	95% CI around estimate
(Intercept)	0.40	0.11	3.75	0.19, 0.61
Informal	0.06	0.01	6.99	0.05, 0.08
Visual appeal	0.50	0.01	60.35	0.48, 0.52
Naturalness	0.10	0.01	9.24	0.07, 0.12
**Random effects**				
Group	Name	Variance	*SD*	95% CI around SD
Participants (*N* = 295)	(Intercept)	1.03	1.01	0.93, 1.11
Image (*N* = 40)	(Intercept)	0.30	0.55	0.44, 0.71
Residual		3.21	1.79	1.77, 1.81
BIC = 48211.2				
**INDIVIDUAL DIFFERENCES MODEL (EXPLORATORY)**				
**Fixed effects**				
Name	Estimate	*SE*	*t*-value	95% CI around estimate
(Intercept)	0.35	0.13	2.67	0.09, 0.61
Informal	0.10	0.01	8.87	0.08, 0.13
Visual appeal	0.49	0.01	59.14	0.48, 0.51
Naturalness	0.09	0.01	8.75	0.07, 0.11
Age	−0.03	0.06	−0.54	−0.15, 0.09
Sex (male)	0.04	0.122	0.33	−0.20, 0.28
Education (no degree)	0.08	0.13	0.63	−0.17, 0.33
Income	−0.06	0.06	−0.98	−0.19, 0.06
Residential experience	0.20	0.06	3.23	0.08, 0.32
Informal x age	0.02	0.01	4.26	0.01, 0.04
Informal × sex (male)	−0.07	0.01	−5.85	−0.09, −0.05
Informal × education (no degree)	−0.03	0.01	−2.30	−0.05, −0.004
Informal × income	−0.01	0.01	−2.27	−0.03, −0.002
Informal × residential experience	−0.0003	0.01	−0.06	−0.01, 0.01
**Random effects**				
Group	Name	Variance	*SD*	95% CI around SD
Participants (*N* = 292)	(Intercept)	0.97	0.99	0.90, 1.08
Image (*N* = 40)	(Intercept)	0.31	0.56	0.45, 0.72
Residual		3.17	1.78	1.76, 1.81
BIC = 47684.1				

### Mediating role of visual appeal

To test whether visual appeal mediated the relationship between informality and perceived restorative potential, a series of linear mixed effects models using maximum likelihood estimation were tested. For all models, participant and image were included as random effects, an unstructured covariance structure was assumed, and variables were logit-transformed. First, it was established that informality significantly predicted perceived restorative potential, *b* = 0.15, *SE* = 0.01, 95% CI [0.13, 0.17], *t* = 15.20, *p* < 0.001. Second, it was confirmed that informality significantly predicted visual appeal, *b* = 0.13, *SE* = 0.01, 95% CI [0.11, 0.15], *t* = 13.76, *p* < 0.001. Third, visual appeal predicted perceived restorative potential, while controlling for informality, *b* = 0.51, *SE* = 0.01, 95% CI [0.49, 0.52], *t* = 61.12, *p* < 0.001. Crucially, after controlling for visual appeal, the relationship between informality and perceived restorative potential was reduced, *b*′ = 0.09, *SE* = 0.01, 95% CI: [0.07, 0.11], *t* = 10.14, *p* < 0.001. Visual appeal partially mediated the relationship between informality and perceived restorative potential. An opposing mediation model was tested to determine whether informality mediates the relationship between visual appeal and perceived restoration. However, this model did not significantly reduce the variance explained by visual appeal (*b* = 0.51, *b*′ = 0.51). Supplemental information regarding mediational analyses can be viewed at https://osf.io/za8q7/.

### Exploratory analysis of individual differences

We conducted an exploratory analysis to estimate if demographic characteristics moderate the relationship between garden design informality and perceived restorative potential. We fit a linear mixed effects model predicting perceived restorative potential from three Level 1 predictor variables of visual appeal, naturalness, and informality, and from five Level 2 predictor variables of participants' self-reported age, sex (male or female), income, education (degree or no degree), and residential experience (population for current county of residence). We included all main effects and all interactions between each Level 2 predictor variable and informality. Perceived restorative potential, visual appeal, naturalness, and informality were logit-transformed. Age, income, and population were centered on the grand mean. We assumed an unstructured covariance structure. Three participants were missing population data and were excluded from the data analysis (*N* = 292).

All Level 1 predictor variables continued to predict variability in perceived restorative potential after accounting for individual differences (see Table 3). Participants currently living in more populated areas reported higher perceived restorative potential ratings than participants currently living in less populated areas.

All Level 2 predictor variables, with the exception of residential experience, significantly moderated the relationship between informality and perceived restorative potential. Older adults and low income participants showed a stronger positive relationship between informality and perceived restorative potential compared to younger adults and higher income participants. Men and participants without a college degree showed a weaker relationship between informality and perceived restorative potential.

## Discussion

The data from our study indicate that people are sensitive to substantive differences between more informal gardens and more formal gardens in regards to perceived restorative potential. Participants perceived gardens to have higher restorative potential when they were visually appealing, had more informal design elements, and contained more natural than built elements. We also explored several potential variables that mediate or moderate the relationship between informality and perceived restorative potential, which are discussed in turn.

### The role of visual appeal

Perceived visual appeal accounted for the most variability in perceived restorative potential. Visual appeal also partially mediated the relationship between informality and perceived restorative potential. That is, more informal gardens were perceived to be more restorative, in part because they were perceived to be more visually appealing than more formal gardens. However, the role of visual appeal in the perceived restorative potential process is still unclear. For example, do people rely on a similar set of environmental features to evaluate visual appeal? If so, what are those features and can they be added systematically to traditionally unappealing settings to increase visual appeal and perceived restorative potential? Future studies can either control for visual appeal across different environment types or systematically vary aspects of the environment to determine their individual effects on visual appeal and perceived restorative potential. Visual appeal does not account for all of the variance in perceived restorative potential so additional unexplored factors must be weighted when people evaluate environments.

### Individual differences

The random effect parameter for participants in our mixed effects model indicated significant variability among participants. Though we did not predict specific moderating relationships of individual differences on perceived restorative potential, we did conduct an exploratory analysis on the demographic data that we collected before the image rating task. Older adults and lower income participants showed a stronger positive relationship between informality and perceived restorative potential compared to younger adults and higher income participants. Men and participants without a college degree showed a weaker relationship between informality and perceived restorative potential. These results are somewhat consistent with a previous study showing that men, younger adults, and higher income participants showed a weaker relationship between naturalness and perceived restorative potential (Twedt et al., under review).

While we can only speculate on the reasons for these moderating effects, theories of place attachment and place identity point to the idea that people have attachments to particular places that resonate with their identities (Korpela and Hartig, 1996; Korpela et al., 2001). Settings for which people have place attachments may be visited to self-regulate following a stressful event. Wilkie and Stavridou (2013) found that people with a preference for urban settings perceived both natural and urban environments to be equal in restorative potential as opposed to people with a preference for natural settings. People who have a preference for urban settings may judge formal gardens to be more restorative than or equally restorative to informal gardens because more built elements are typically present in formal gardens.

Additional moderating factors may also predict variability across individuals, such as the degree to which people currently seek a restorative experience. Individuals who are currently fatigued, mentally exhausted, or experiencing negative affect may judge gardens differently than people whose resources are not currently depleted. This expected result follows several studies in which perceived restorative potential of natural, built, and mixed natural-built settings depended on individuals' current or imagined needs for restoration (Korpela, 2003; Staats et al., 2003; Hartig and Staats, 2006; Twedt et al., under review).

A promising avenue for future research is to systematically test whether different types of people use different criteria for evaluating the perceived restorative potential of environments. A few potential reasons include variability in access to certain types of spaces, aesthetic preferences, and feeling inclusive within a given space. Also intriguing is the possibility that certain cultures may value particular types of gardens differently than other cultures, influencing the way in which gardens are evaluated.

### Design implications

A primary emphasis within the restorative environment literature is the mental and emotional health benefits of natural environments. A brief walk in nature, compared to a brief walk in an urban setting, has been linked to improved directed-attention abilities, better working memory performance, decreased anxiety and rumination, and lower negative affect (Berman et al., 2008; Bratman et al., 2015a,b). In a recent meta-analysis of over 30 experiments, McMahan and Estes (2015) found that spending time in nature had a consistent effect of increasing positive affect and of decreasing negative affect. Interacting with nature has been shown to improve mood and cognitive performance in individuals diagnosed with major depressive disorder (Berman et al., 2012), and mere proximity to green spaces is associated with less symptomology for depression, anxiety, and stress (Beyer et al., 2014).

Given the potential for natural environments to benefit mental and emotional well-being, it is desirable for individuals to have easy access to restorative settings, particularly when the need for restoration is high. Incorporating a garden into a building design or into an urban landscape can be a way to provide individuals with restorative experiences without needing to travel far from their everyday environments.

Hospitals and elder care facilities often incorporate healing or therapeutic gardens into building design. A healing garden is a space that contains ample vegetation and other elements addressing the needs of patients in a particular medical context. It is designed to provide therapeutic benefits to hospital staff and visitors as well (Cooper-Marcus and Barnes, 1999; Ulrich, 1999). Previous research on healing gardens has focused on stress reduction. Ulrich (1999) suggests that a successful healing garden has the potential to reduce stress and improve health outcomes if it fosters social support, exercise, access to nature, sense of control and privacy, and a sense of security (Cooper-Marcus and Sachs, 2014).

Most of the research to date on gardens in healthcare facilities has been of a qualitative nature, involving questionnaires and more anecdotal post-occupancy evaluations of built gardens. Design principles for gardens for dementia sufferers, as well as for end-of-life care, elder care, and HIV/AIDS patients, have been extensively documented, using predominantly qualitative methodologies (Zeisel and Tyson, 1999; Cooper-Marcus and Sachs, 2014). More quantitative work is needed, focusing on physiological measurements of health outcomes. This will allow more precise focus on the multisensory nature of gardens and restorative experiences involving sound, touch, smell, and taste, in addition to sight. It will also allow for comparison between perceived restoration and actual restoration, which will indicate how well people can perceive that certain spaces might be better for their health during certain circumstances. Also more studies of gardens in other healthcare contexts where patients experience high stress, such as autism and post-traumatic stress disorder, are called for (Herbert, 2003; Detweiler et al., 2010).

## Conclusions

The current study shows that visual appeal, informal design elements, and naturalness make a garden optimal for restoration, from the perspective of potential visitors. More informal gardens, as opposed to more formal gardens, are perceived to be more visually appealing and more natural, making them ideally suited for restorative experiences. The influence of judged visual appeal, however, suggests that it might be possible for the perceived restorative potential of formal gardens to be increased if visually appealing elements are added.

Although informal gardens are perceived to be more natural than formal gardens, this naturalness was the product of human design. Because gardens are built spaces, landscape architects, architects, and other designers can incorporate findings such as ours into their designs to create restorative, natural-looking spaces.

## Author contributions

ET, RR, and DP contributed to the conception and design of the research. ET acquired, analyzed, and interpreted the data. ET, RR, and DP wrote the paper. All authors provided final approval of the version submitted for review.

### Conflict of interest statement

The authors declare that the research was conducted in the absence of any commercial or financial relationships that could be construed as a potential conflict of interest.
